# Exophytic gastrointestinal stromal tumor with cystic changes: A case report

**DOI:** 10.3892/ol.2014.1954

**Published:** 2014-03-07

**Authors:** CHUN-CHAO ZHU, YE LIU, GANG ZHAO

**Affiliations:** Department of Gastrointestinal Surgery, Ren Ji Hospital, School of Medicine, Shanghai Jiao Tong University, Shanghai 200127, P.R. China

**Keywords:** exophytic gastrointestinal stromal tumor, cystic change, prognosis

## Abstract

Gastrointestinal stromal tumor (GIST) is the most common type of mesenchymal tumor in the gastrointestinal tract. A large tumor size often means a poor prognosis. This report presents a case of a large exophytic GIST with cyst change, for which the outcome of favourable prognosis was unexpected. A 78-year-old male presented with abdominal distension and a poor appetite, and was primarily diagnosed with a pancreatic mass. Abdominal ultrasonography, computed tomography and magnetic resonance imaging revealed a tumor in the body of the pancreas, which was closely attached to the gastric wall. Surgery was performed to excise the tumor. The tumor originated from the gastric cells and was ~17×15×16 cm in size. A diagnosis of GIST was confirmed by histomorphological and immunohistochemical findings. According to the postoperative findings, the tumor was classified to be in the high-risk group, for which the suggested treatment is imatinib. However, the patient was not treated with imatinib and, three years following surgery, the patient is alive with no evidence of tumor recurrence.

## Introduction

Gastrointestinal stromal tumor (GIST) is the most common type of mesenchymal tumor in the gastrointestinal (GI) tract, with a disease incidence of 10–20 per million individuals worldwide (1–3). GIST can occur in any region of the digestive tract and the incidence of GIST in the stomach, small intestine, large intestine and esophagus is reported to be 60–70, 20–30, 18.1 and 1.4%, respectively. According to the tumor location, GISTs are classified as endoluminal, exoluminal, intramural and mixed types (4). Immunohistochemical findings and the ultramicrostructure of GIST cells are similar to those of Cajal cells, which are autonomous nerve-related GI pacemaker cells that regulate intestinal motility (5,6). On diagnosis, immunohistochemical analysis revealed the presence of cluster of differentiation (CD)117-positive and CD34-positive/negative tumor cells. The most typical characteristic of malignancy is infiltration of neighboring organs or lymph node metastasis. Infiltration of the lamina propria mucosae or the muscular layer is an important indicator for the diagnosis of malignant GIST. In addition, manifestation of the malignancy includes a large tumor size (diameter of >5 cm for gastric tumors and >4 cm for small intestine tumors), obvious mitosis count [>5/50 high-power fields (HPFs)] (7), high density of cells, infiltration of the lamina propria mucosae, presence of coagulative tumor necrosis (8), high Ki-labeling index (>5%) (9,10), recurrence and metastasis. Patient provided written informed consent.

## Case report

**Figure 1:**
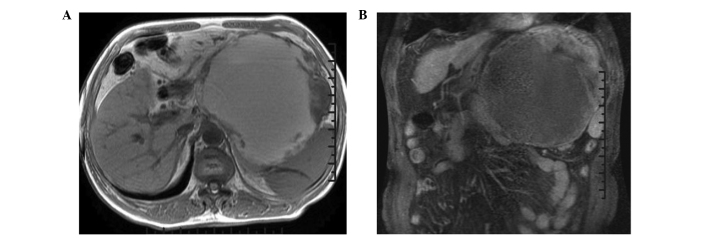
(A) Magnetic resonance imaging revealed a large cystic-solid mass of 17×15×16 cm in size that had grown into the lesser omental bursa. (B) Coronal plane shows the stomach had changed shape due to compression and the mass was closely attached to the gastric wall.

A 78-year-old man presenting with abdominal distension and a poor appetite was diagnosed with a pancreatic mass and referred to the Renji Hospital (Shanghai, China) for treatment on November 10, 2008. Hematological testing showed that tumor antigen and routine blood test results were normal. Abdominal ultrasonography revealed a hypoechoic mass with an uneven irregular surface and a clear boundary in the middle upper abdomen; the tumor showed mixed echogenicity. Magnetic resonance imaging (MRI) revealed a large cystic-solid mass that had grown into the lesser omental bursa (Fig. 1A) and the stomach had changed shape due to compression (Fig. 1B). The observed mass was likened to pancreatic cystadenocarcinoma or canceration of cystadenoma. Computed tomography (CT) revealed a tumor in the body of the pancreas, which was closely attached to the gastric wall. Surgical treatment was performed to excise the tumor on September 15, 2008. The mass was ~17×15×16 cm in size with a thick wall that was completely attached to gastric wall tissue. The tumor was multicystic and partly solid, contained watery brown fluid, compressed the pancreas and had a smooth outer surface. In addition, an ulcer measuring ~0.5 cm in diameter was observed where the mass adhered to the gastric wall. The mass, part of the stomach, the spleen and the greater omentum were surgically removed.

**Figure 2:**
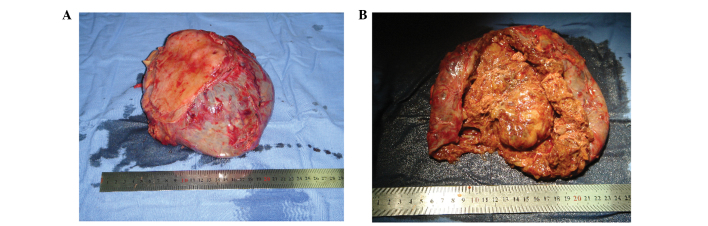
(A) The resected tumor was a well-circumscribed mass measuring 15×17×13 cm in size. An ulcer was found on the resected gastric wall where it was attached to the tumor. (B) The solid portion of the mass was pink-gray in color and was soft with a scaly appearance.

**Figure 3:**
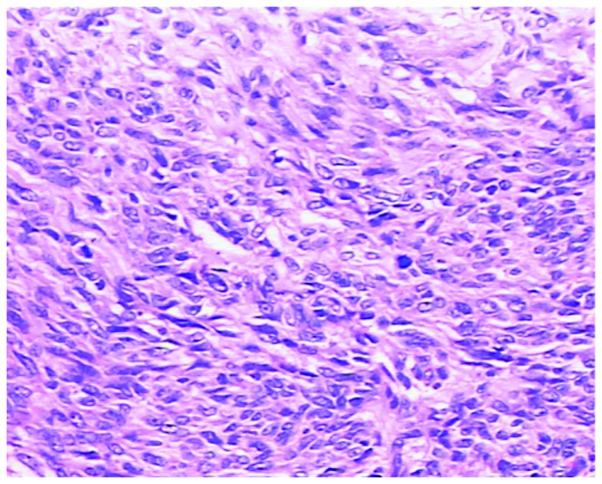
Microscopically, the tumor cells were epithelioid or spindle-shaped, arranged in an ill-defined fascicular pattern (stain, hematoxylin and eosin; magnification, ×200).

**Figure 4:**
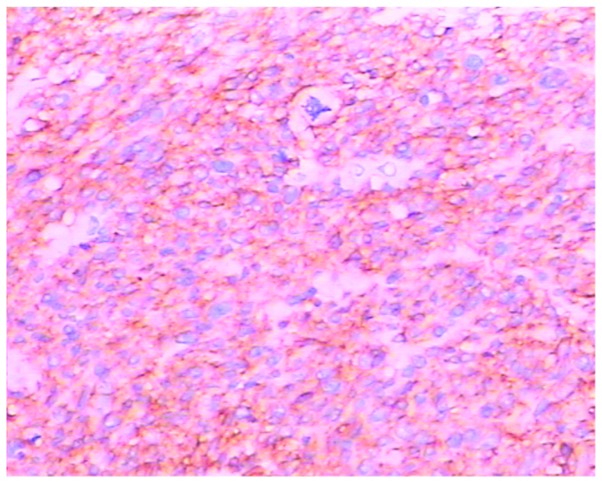
Immunohistochemical analysis revealed that the tumor cells were cluster of differentiation 117-positive (magnification, ×200).

The resected tumor was a well-circumscribed mass, measuring 15×17×13 cm in size. An ulcer was found on the resected gastric wall where it was attached to the tumor (Fig. 2A). The solid portion was pink-gray in color, soft and had a scaly appearance (Fig. 2B). Microscopically, the tumor cells were epithelioid or spindle-shaped and arranged in an ill-defined fascicular pattern. Pathologically, the mitosis count was >10/50 HPFs (Fig. 3). Immunohistochemical staining revealed that the tumor cells were CD34-positive, CD117-positive (Fig. 4), Ki67-positive, S-100-positive/negative, smooth muscle actin-negative and p53-negative. Thus, the final diagnosis was GIST that was highly malignant.

The patient was discharged from hospital 14 days following surgery and was not treated with imatinib (Gleevec^®^; Novartis AG, Basel, Switzerland) due to financial reasons. Follow-up revealed that the patient is alive three years after surgery with no evidence of tumor recurrence.

## Discussion

GIST typically appears as a regular, soft, solid mass and rarely presents with cystic changes as the main clinical manifestation. Exophytic stromal tumors with cystic changes have been previously reported; however, large cystic mesenchymal tumors are rarely observed. As the number of available studies on exophytic stromal tumors with cystic degeneration are currently limited, almost all authors suggest that, during preoperative diagnosis, these masses may be mistaken to have derived from the liver or pancreatic tissue. In our case, the patient presented with abdominal distension and anorexia with no other gastrointestinal symptoms, such as vomiting or melena. Upper gastrointestinal barium meal imaging revealed no obvious abnormalities and preoperative MRI and CT suggested the presence of a tumor derived from the pancreatic body and tail. Therefore, the tumor was misdiagnosed as a pancreatic body and tail tumor. The use of ultrasound-guided endoscopy may have provided further diagnostic evidence.

In the present case, the size of the tumor was >5 cm, which is the standard size used to discriminate benignity from malignancy for GIST. In accordance with the current criterion for benignity and malignancy in GIST (11), the present case was classified into the malignancy group, a high-risk group with a poor prognosis. However, in the absence of imatinib treatment, the results of the follow-up examinations were unexpected, as no evidence of tumor recurrence or metastasis was reported three years after surgery. Thus, the current criterion for the benignity and malignancy of GIST may be debatable. A review of the currently available literature suggests that in cases of cystic stromal tumors, tumor size is not a true indicator of benignity or malignancy. However, the solid part of the tumor may be included in the criteria for indicating benignity or malignancy. Wang *et al* (12) suggested that the size of the tumor is difficult to determine objectively. In cases of GIST with cystic degeneration, the larger the area of the cystic component, the lower the objectivity in determining tumor size (13). In addition, Kim *et al* (14) found that among cases of GIST with a diameter of <5 cm, almost half of the tumors showed internal bleeding, necrosis or cystic degeneration and the prognosis was not necessarily associated with tumor size. GIST with cystic changes may be observed in the following situations: i) primary cystic GIST, in which the main structure comprises cystic tissue with a pseudocapsule, rarely invading the surrounding organs; ii) malignant GIST with cystic degeneration, caused by rapid growth of the tumor, which due to insufficiency of the internal blood supply results in necrosis and liquefaction; iii) when the tumor metastasizes to the liver and pancreas, the metastatic lesion is always cystic in nature, often confused with liver cysts and pancreatic cysts; and iv) on treatment with imatinib, malignant GISTs show cystic degeneration (15).

On reviewing this case and the currently available literature, we suggest that further pathological investigations of cystic GIST are required to avoid potentially excessive or inappropriate administration of imatinib.
